# Increased HIV Testing among Men Who Have Sex with Men from 2008 to 2012, Nanjing, China

**DOI:** 10.1371/journal.pone.0154466

**Published:** 2016-04-25

**Authors:** Hongjing Yan, Jianjun Li, H. Fisher Raymond, Xiping Huan, Wenhui Guan, Haiyang Hu, Haitao Yang, Willi McFarland, Chongyi Wei

**Affiliations:** 1 Jiangsu Provincial Centers for Disease Control and Prevention, Nanjing, Jiangsu, China; 2 Department of Epidemiology and Biostatistics, University of California San Francisco, San Francisco, California, United States of America; 3 San Francisco Department of Public Health, San Francisco, California, United States of America; The University of New South Wales, AUSTRALIA

## Abstract

**Background:**

HIV testing is the first point of HIV treatment entry for HIV-infected individuals and an avenue to engage persons at risk in prevention. In China, where the prevalence of HIV among men who have sex with men (MSM) has been rising over the last decade, uptake of HIV testing has been low.

**Methods:**

We examined changes in HIV testing in the preceding 12 months through two cross-sectional surveys conducted among MSM in Nanjing, Jiangsu province, China in 2008 and 2012. Respondent-driven sampling (RDS) was used to recruit participants. Questionnaire interviews and venous blood were collected to measure HIV testing, risk behaviors, and prevalence of HIV, syphilis, and HSV-2.

**Results:**

A total of 430 and 589 MSM were surveyed in 2008 and 2012, respectively, with comparable samples in each round with respect to demographic characteristics. HIV testing in the past 12 months increased significantly from 20.1% (95% CI 13.3–26.8) in 2008 to 46.0% (95% CI 39.3–51.4, *p* < 0.001) in 2012. HIV prevalence was stable, at 6.6% (95% CI 2.5–11.3) in 2008 and 10.1% (95% CI 6.6–13.9, *p* = 0.240) in 2012, as was syphilis (14.3% in 2008 vs. 9.9% in 2012, *p* = 0.240). HSV-2 prevalence (18.6% in 2008 vs. 10.2% in 2012, *p* = 0.040) and self-reported STI in the last year (24.3% in 2008 vs. 14.3% in 2012, *p* = 0.020) significantly decreased. Changes in reported sexual behaviors were mixed and the profiles of who did and did not test varied between 2008 and 2012.

**Conclusions:**

HIV testing uptake more than doubled among MSM in Nanjing from 2008 to 2012 –a period of massive promotion and scale up of testing programs for MSM. However, additional efforts are still needed to further increase the proportion of men being not only tested but also undergoing repeat testing if they engage in continued risk taking behavior.

## Introduction

An expanding HIV epidemic among men who have sex with men (MSM) in China has been widely and amply documented over the last decade [1–4]. A meta-analysis of MSM studies throughout China found that HIV prevalence increased from 0.6% in 2003 to 7.4% in 2009 [1]. Recent cohort studies of MSM in diverse cities such as Beijing, Guangzhou, and Shenyang measured HIV incidence greater than 5% [2–4]. Other indicators of the potential for HIV transmission include high risk behaviors and prevalence of syphilis, herpes simplex virus type 2 (HSV-2) and other sexually transmitted infections (STIs) [5–9]. For example, a meta-analysis of syphilis serological surveys found prevalence increased from 6.8% in 2003/04 to 13.5% in 2007/08 [5]. Meanwhile, rates of testing for HIV among Chinese MSM are low [10].

In response to the escalating HIV epidemic among MSM, the Chinese government developed national guidelines on HIV prevention specifically for MSM in 2006 [11]. A minimum HIV prevention package was defined and implemented nation-wide, including peer education, condom promotion, HIV testing and counseling, STI treatment, providing social support and clinical care for people living with HIV/AIDS (PLWHA). In 2011, to strengthen efforts among key affected populations, such as MSM, the State Council of China implemented the “Five Expands, Six Strengthens” approach for HIV prevention and control [12]. The program centered on the expansion of HIV testing services with scaling up of care, support, and treatment for HIV-infected individuals. For example, over 9,000 HIV voluntary testing and counseling sites were established across the country to offer free HIV testing and care and treatment services for diagnosed cases [13].

Nanjing, the capital of Jiangsu province, is located in eastern China. The residential population is 6.36 million and the estimated migrant population is 1.75 million [14]. As the economic and cultural center of the province, Nanjing has a sizable population of gay men and other MSM, estimated to be 20,000 [15]. Prospective cohort studies of MSM in Nanjing reported an HIV incidence of 5.12 per 100 person-years, and an incidence of syphilis of 7.58 per 100 person-years [16, 17]. To track the HIV epidemic among MSM, Nanjing is part of a system of 108 national sentinel surveillance sites that conduct serial cross-sectional surveys annually using a venue- and referral- based sampling methodology [18]. However, an external evaluation of the system by the US CDC Global AIDS Program China Office found that the relatively small sample sizes and convenience sampling approach may not accurately reflect changes in the epidemic and the impact of intervention efforts over time [18].

To fill the gap with more accurate data on MSM, we implemented respondent-driven sampling (RDS) in two cross-sectional bio-behavioral surveys in Nanjing in 2008 and 2012. RDS is designed to obtain a more representative sample through long recruitment chains, recruitment limits, and statistically adjusting for the inherent biases in how persons are connected in and recruited from their social networks [19]. The approach has been widely used for surveys of MSM worldwide, although less often described for multiple rounds of surveys [9, 20]. Because the two waves straddle a period of massive efforts to increase HIV testing among MSM, this paper aims to evaluate the effect of these efforts on uptake of HIV testing within this population. In addition to HIV testing uptake, we assess changes in other prevention indicators, including HIV, syphilis, and HSV-2 prevalence; condom use; and other risk behaviors since the implementation of the four-year national comprehensive HIV prevention package for MSM.

## Methods

### Study Design and Participant Eligibility

We conducted two cross-sectional survey studies of MSM in Nanjing, China in 2008 and in 2012. The two surveys strictly followed procedures based on the RDS methodology, implemented at the same interview site—the Jiangsu provincial HIV testing and counseling clinic, a designated MSM sentinel surveillance site since 2006 and where a majority of HIV testing for MSM in the city is conducted, by the same research team. Administered questionnaires of 2012 covered all information of 2008 and same laboratory tests, and used the same eligibility criteria. Participants were 18 years old or older, were male, reported engaging in anal or oral sex with another man in the past year, and were willing to be tested for HIV, syphilis and HSV-2.

### Sampling and Recruitment

RDS was used to recruit MSM in both surveys. We have described the standardized methods previously [16, 21] and paraphrase ourselves here. In 2008, recruitment chains began with nine “seeds” who initiate peer-referrals: 2 from gay saunas, 2 from gay bars, 2 via the internet, 1 from a park, and 2 via peer referral. In 2012, ten seeds were recruited: 2 from gay saunas, 2 from gay bars, 2 via the internet, 2 from parks, and 2 via peer referral. Each seed underwent consent procedures, completed a face-to-face interview, provided blood specimens for HIV, syphilis, and HSV-2 testing, and received 3 recruitment coupons to recruit up to 3 MSM peers (i.e., recruits). Each coupon had an expiration date, a unique study number, information about the testing site, and a contact number for inquiries. Recruits who presented with a valid coupon and met eligibility criteria underwent the same process as his recruiter to complete the survey and provide specimens. These recruits then became recruiters, and were given 3 coupons to recruit their peers. These recruitment chains continued until equilibrium and the sample size was achieved. “Equilibrium” in RDS is when the composition of the sample with respect to key variables does not substantially change with subsequent waves of recruitment. It is the point of stability in the sampling process such that suggests all groups who are going to be reached are likely have been reached, that continuation of recruitment is unlikely to change estimates, and that sufficient data have been obtained to calculate survey weights (i.e., differential probability of inclusion) and homophily (i.e., clustering of similar characteristics between recruiter and recruits). The formative assessment and the prior surveys [16, 21] determined which variables were key measures to ensure equilibrium (i.e., that were likely to denote different sub-networks). These variables were marital status, residence, education level, and syphilis prevalence. They were identified as characteristics that influence recruitment and represent the diversity of the population. Achieving equilibrium on these variables was posited as indicative of a successful RDS recruitment. In 2008, equilibrium was reached at the 9th wave with all four key variables and recruitment continued through 13 waves of referrals to include 430 participants. In 2012, equilibrium was reached at the 8th wave and 589 participates were recruited through 13 waves of referrals. When the sample size was reached, several waves after equilibrium, recruitment was tapered by reducing recruitment coupons from 3 to 2 to 1 to 0. Given that equilibrium was achieved several waves prior to tapering recruitment, the impact on the sample was projected to be minimal. The proportion of the coupons that ultimate returned was 42% in 2008, and 38% in 2012. This is typical of RDS surveys [20].

### Measures

A brief standardized questionnaire was administered face-to-face in a private room by trained research staff to collect socio-demographic information including age, marital status, official residential status (houkou), education attainment, monthly income, and sexual orientation. Risk-related measures included number of male sex partners, condomless anal sex with men, whether participants exchanged sex for money, sex with women, HIV testing in the past 12 months, and history of STIs in the past year.

A venous blood sample (5 ml) was obtained for rapid HIV testing and later syphilis and HSV-2 testing. HIV rapid tests (Acon Biotech, Hangzhou, China) were conducted for HIV screening and positive results were retested with an additional rapid test (Serodia HIV, Fujirebio, Japan), and Western Blot confirmatory tests (HIVBLOT 2.2, Genelabs, Singapore) were conducted for repeated positives. Syphilis was tested using the Raid Plasma Regain (RPR, Beijing WanTai Biological Pharmacy Enterprise, Beijing, China) for screening, and positive results were confirmed by Treponema Pallidum Particle Agglutination assay (Livzon Group, Zhuhai, China). HSV-2 was tested using Herpes Select 2 ELISA assay (Focus Diagnostics, USA). Participants received pre- and post- HIV test counseling at the clinic. HIV results were disclosed to participants onsite by certified counselors. Those who preliminarily screened HIV positive were asked to return in person to receive repeat HIV testing and confirmatory testing. Referral services were offered by counselors if confirmed HIV-positive. Syphilis and HSV-2 screening results were returned two weeks later by research staff and relevant referral services were provided to those whom tested positive for STIs. All assays and algorithms were approved by China’s national guidelines.

Participants were given a box of free condoms, lubricant and prepaid phone cards (30RMB: 5 USD in 2008; 50RMB: 8USD in 2012) as incentives for completing the survey and providing specimens. The proportion of men who returned for confirmatory testing in 2008 was 85%, and 90% in 2012.

### Statistical Analysis

Data were analyzed using the Respondent-Driven Sampling Analysis Tool (RDSAT) version 5.6 (available for free online, http://respondentdrivensampling.org) to produce population estimates. The method adjusts the crude sample estimates to reflect the composition of the target population based on tracking who recruited whom and the relative sizes of participants’ networks [19]. Because RDSAT does not directly perform statistical testing, adjusted 95% CIs were used to interpret differences within and between survey years. The amount of overlap of the confidence intervals can be tested for statistical significance using the Z-test [22]. To identify independent correlates of HIV testing in the past year, we imported data into SPSS 20.0 to conduct multivariate logistic regression analysis. Statistical significance was defined by a p-value < 0.05. RDS does result in design effects that are adjusted for in RDSAT [23]. The generated population estimates include 95% confidence intervals based on corrected standard errors. Our statistical test, the Z-test, is based on the distribution of overlap of the adjusted confidence intervals and therefore the comparison of estimates between survey waves accounts for the design effects. For the multivariate analysis, RDSAT exports individual weights that adjust the standard errors in the logistic regression analysis.

### Ethical Approval

The 2008 study was reviewed and approved by the Ethics Committee of the Chinese Central for Disease Control and Prevention and the 2012 study was reviewed and approved by the Institutional Review Board of Jiangsu provincial CDC. Written informed consent was obtained from all participants.

## Results

A total of 430 MSM completed the survey from May to August in 2008, and 589 from September to December in 2012. Recruitment chains progressed up to 13 waves in 2008 and 9 waves in 2012. Equilibrium was reached in both studies on the four variables tracked (marital status, residence, education level, and syphilis prevalence). The composition of the samples and their corresponding population-adjusted measures were comparable in the two survey rounds (Table 1), including age, education, income, residency, and sexual orientation. Significantly more MSM were divorced or widowed in 2012 compared to 2008 (19.8% vs. 7.8%, respectively, *p* < 0.001) and fewer were single (55.4% vs. 65.2%, respectively, *p* = 0.040).

**Table 1:** Crude and population-adjusted characteristics of men who have sex with men (MSM) in two respondent-driven sampling (RDS) surveys, Nanjing, China, 2008 and 2012.

	2008 (N = 430)		2012 (N = 589)			
	Crude % (n)	Adjusted % (95% CI)	Crude % (n)	Adjusted % (95% CI)	Z-statistic	p-value
Age under 30 years:	66.3 (285)	58.1 (48.8–67.7)	58.7 (346)	55.8 (49.5–61.6)	0.402	0.680
Less than high school education	33.0 (142)	40.5 (31.1–49.9)	42.1 (248)	47.5 (41.6–53.8)	1.224	0.220
Monthly income <2,000 RMB^1^	55.8 (240)	51.9 (42.8–59.9)	36.0 (212)	41.7 (35.9–47.5)	1.935	0.060
Residency (hukou) in Nanjing	58.4 (251)	47.6 (39.6–55.7)	49.9 (294)	47.3 (41.5–53.2)	0.059	0.960
Marital status:						
Single	76.3 (328)	65.2 (57.2–73.7)	58.7 (346)	55.4 (50.2–59.9)	2.007	0.040
Married	18.8 (81)	27.0 (19.6–34.1)	19.7 (116)	24.7 (20.5–29.1)	0.535	0.600
Divorced or widowed	4.9 (21)	7.8 (3.8–12.0)	21.6 (127)	19.8 (16.7–24.0)	4.285	<0.001
Sexual orientation:						
Gay	56.3 (242)	54.2 (47.9–61.1)	60.6 (357)	49.4 (43.1–55.0)	1.059	0.280
Heterosexual	2.3 (10)	1.9 (0.6–3.7)	1.4 (8)	3.8 (1.3–6.9)	1.168	0.240
Bisexual / unsure	41.4 (178)	43.9 (37.2–49.8)	38.0 (224)	46.8 (36.8–58.2)	0.458	0.640

^**1**^1 USD ≈ 6 RMB.

Testing for HIV in the last 12 months significantly increased to 46.0% (95% CI 39.3–51.4) in 2012 from 20.1% (95% CI 13.3–26.8, *p* < 0.001) in 2008 (Table 2). HIV prevalence was comparatively stable, with 10.1% (95% CI 6.6–13.9) testing positive in 2012 compared to 6.6% (95% CI 2.5–11.3) in 2008 (*p* = 0.240). Syphilis seropositivity also showed no significant change (9.9% in 2012 vs. 14.3% in 2008, *p* = 0.240). HSV-2 seroprevalence (10.2% in 2012 vs. 18.6% in 2008, *p* = 0.040) and self-reported STI in the last year (14.3% in 2012 vs. 24.3% in 2008, *p* = 0.020) was significantly lower in 2012 compared to 2008. Changes in reported sexual behaviors were mixed. While there was a significant increase in reporting condom use at last sex with a man (66.3% in 2012 vs. 45.1% in 2008, *p* < 0.001), reporting any condomless anal sex in the last six months was stable (61.2% in 2012 and 62.1% in 2008, *p* = 0.840). Buying sex from a man in the last six months increased to 7.6% in 2012 from 2.2% in 2008 (*p* < 0.001). Multiple partners and selling sex in the last six months did not significantly change.

**Table 2:** Crude and population-adjusted prevalence of HIV, sexually transmitted infections (STI), and risk behaviors in two respondent-driven sampling (RDS) surveys of men who have sex with men, Nanjing, China, 2008 and 2012.

	2008 (N = 430)		2012 (N = 589)			
	Crude % (n)	Adjusted % (95% CI)	Crude % (n)	Adjusted % (95% CI)	Z-statistic	p-value
Tested for HIV, last 12 months	22.3 (96)	20.1 (13.3–26.8)	48.4 (285)	46.0 (39.3–51.4)	5.600	<0.001
HIV prevalence	4.7 (20)	6.6 (2.5–11.3)	9.2 (54)	10.1 (6.6–13.9)	1.200	0.240
Syphilis prevalence	12.6 (54)	14.3 (8.6–21.9)	11.4 (67)	9.9 (6.9–13.8)	1.151	0.240
HSV-2 prevalence	16.0 (69)	18.6 (12.4–26.6)	11.5 (68)	10.2 (7.1–13.7)	2.103	0.040
Self-reported STI, last 12 months	25.6 (110)	24.3 (17.9–31.9)	14.1 (83)	14.3 (10.3–18.7)	2.401	0.020
Condom use last sex with a man	50.2 (216)	45.1 (37.4–51.3)	58.9 (347)	66.3 (59.8–72.9)	4.351	<0.001
Condomless anal sex, last 6 months	60.5 (260)	62.1 (55.1–69.6)	62.8 (370)	61.2 (55.4–66.5)	0.193	0.840
≥ 2 sexual partners, last 6 months	59.1 (254)	56.0 (48.2–63.9)	59.1 (348)	48.8 (42.3–54.0)	1.441	0.140
Sold sex to men, last 6 months	5.1 (22)	7.3 (3.1–11.6)	4.6 (27)	6.0 (2.3–7.2)	0.519	0.600
Bought sex from a man, last 6 months	4.4 (19)	2.2 (1.0–5.0)	6.3 (37)	7.6 (3.4–9.3)	2.970	<0.001

Table 3 shows the profiles of MSM who tested for HIV in the last 12 months in 2008 and 2012. In 2008, factors significantly associated with HIV testing were being gay-identified (AOR 2.15, 95% CI 1.22–3.80, *p* = 0.009), being syphilis positive (AOR 2.72, 95% CI 1.42–5.20, *p* = 0.003), being HSV-2 positive (AOR 2.05, 95% CI 1.09–3.86, *p* = 0.026), and self-reporting an STI in the past year (AOR 2.11, 95% CI 1.22–3.67, *p* = 0.008). In 2012, factors significantly associated with HIV testing were having college education or higher (AOR 1.68, 95% CI 1.15–2.45, *p* = 0.007), having a monthly income of less than 2000 RMB (AOR 1.59, 95% CI 1.11–2.28, *p* = 0.012), being married (AOR 1.68, 95% CI 1.09–2.60, *p* = 0.019), being gay-identified (AOR 1.57, 95% CI 1.08–2.27, *p* = 0.017), being HIV negative (AOR 2.95, 95% CI 1.48–5.89, *p* = 0.002), being HSV-2 positive (AOR 2.25, 95% CI 1.22–4.14, *p* = 0.009), and having sold sex to other men in the last six months (AOR 3.19, 95% CI 1.45–7.02, *p* = 0.004).

**Table 3:** Factors associated with HIV testing among MSM in Nanjing, China, 2008 and 2012.

	B	AOR	95% CI	S.E.	Wald	p-value
**2008**						
Sexual orientation						
Bisexual / unsure	Ref	1	--	--	7.06	0.029
Gay	0.77	2.15	1.22–3.80	0.29	6.92	0.009
Heterosexual	0.74	2.09	0.13–34.69	1.43	0.27	0.606
Syphilis positive	1.00	2.72	1.42–5.20	0.33	9.08	0.003
HSV-2 positive	0.72	2.05	1.09–3.86	0.32	4.97	0.026
Self-reported STI, last year	0.75	2.11	1.22–3.67	0.28	7.04	0.008
**2012**						
College education or higher	0.52	1.68	1.15–2.45	0.19	7.17	0.007
Monthly income <2,000 RMB	0.46	1.59	1.11–2.28	0.19	6.28	0.012
Marital status						
Single	Ref	1	--	--	6.86	0.032
Married	0.52	1.68	1.09–2.60	0.22	5.46	0.019
Divorced or widowed	-0.14	0.87	0.54–1.41	0.24	0.31	0.578
Sexual orientation						
Bisexual / unsure	Ref	1	--	--	7.23	0.027
Gay	0.45	1.57	1.08–2.27	0.19	5.66	0.017
Heterosexual	0.81	2.26	0.86–5.92	0.49	2.75	0.098
HIV negative	1.08	2.95	1.48–5.89	0.35	9.40	0.002
HSV-2 positive	0.81	2.25	1.22–4.14	0.31	6.79	0.009
Sold sex to men, last 6 months	1.61	3.19	1.45–7.02	0.40	8.33	0.004

## Discussion

HIV testing more than doubled among MSM in Nanjing from 2008 to 2012, according to the evidence of our surveys. In 2008, only one in five MSM had tested for HIV in the last year, rising to nearly half in 2012. Our RDS methods were identical for the two rounds, implemented by the same team, and theoretically more rigorous than convenience samples in obtaining representative cross-sectional samples of this hard-to-reach population [19, 20]. Moreover, demographic data (with the exception of a change in marital status) were consistent across both surveys. The increase occurred following changes in policies to promote HIV testing as a cornerstone of the national response to the epidemic, massive scale up of more than 9,000 testing sites, and support for MSM community-based organizations (CBOs) to make testing more accessible [11–13]. We note that the profile of MSM more likely to test included gay men in both survey rounds, in line with the promotion of frequent testing for self-identified key populations at higher risk for HIV. In addition, the profile of MSM more likely to test expanded in 2012 to include different demographic groups by education level, income, and marital status. These factors strengthen the inference that there was a real increase in HIV testing overall and in the reach to more diverse groups of MSM in the four-year period.

The increase in HIV testing can have individual health benefits and community prevention impact through referral of persons diagnosed to care, early initiation of ART, and suppression of viral load [24–26]. With support from international cooperative programs on HIV prevention in China, such as the Global Fund and Bill & Melinda Gates Foundation, a growing number of MSM CBOs are capacitated to provide peer-led HIV rapid testing and referrals to local CDCs for confirmatory testing and linkage to care [27–29]. The collaboration of CBO- and government facility- based HIV testing, counseling, and care has become a model for making high quality, MSM-friendly services in China [30].

However, caution must be exercised in attributing the increase in reported HIV testing to particular programs. Other temporal changes are likely to have occurred during the same period, such as increase in HIV treatment, availability of largely unregulated self-tests on the internet, and changes in attitudes towards testing among MSM and the general population. It is also possible that the samples differed between the two waves and that there was not a real increase in testing among MSM. We also acknowledge that these serial cross-sectional surveys do not prove causality; rather they provide some support to the possibility that the increases in HIV testing were real and attributable in part to the programs scale up during the interval.

We recognize other limitations of our study. Foremost, there is no gold standard sampling approach for hidden populations for HIV research and no census of MSM against which to compare our data. Although we believe our methods conformed to RDS theory and practice, and our two survey samples appeared comparable on most demographic variables, we cannot verify that the samples were representative and therefore a valid measure of changes from 2008 to 2012. Second, it is possible that bias may have falsely elevated the levels of self-reported testing in 2012. For example, social desirability may favor saying that one has tested for HIV to a greater degree in 2012 compared to 2008 given the efforts to promote testing. Caution must also be exercised in interpreting changes in other variables as causally linked to the observed increase in HIV testing.

While suggesting a substantial improvement in HIV testing, the two surveys provide a mixed picture on changes in other risk and preventive behaviors among MSM from 2008 to 2012. On the one hand, reported condom use at last sex with a man increased from fewer than half in 2008 to nearly two-thirds in 2012. Self-reported STI in the last year and HSV-2 seropositivity also decreased. On the other hand, any condomless sex with a man, multiple partners, selling sex, and syphilis prevalence showed no significant change; buying sex increased. HIV prevalence was also stable; however, the estimates of 6.6% in 2008 and 10.1% in 2012 must be interpreted in the context of a relentless rise from 0% in 2003 [31] and an increase in treatment and survival with HIV. Nonetheless, our data do suggest the focus of promoting regular, repeat testing among MSM through CBO and governmental programs can impact its primary target. In fact, even after the massive efforts by the Chinese government to test more MSM, less than 50% of these men were tested in the past year. This sends a clear message that additional efforts are still needed to further increase the proportion of MSM being not only tested but also undergoing repeat testing if they engage in continued risk taking behaviors. Such efforts include a recently established domestic fund to support CBOs’ increased involvement in HIV/AIDS prevention by the government [32]. CBOs are also piloting innovative strategies to reach and test more MSM including outreach on the Internet and providing low-cost HIV self-testing kits. These efforts and strategies should be supported and scaled up on a timely manner to reduce HIV infections among Chinese MSM.
